# Effects of Oxygen Limitation on the Biosynthesis of Photo Pigments in the Red Microalgae *Galdieria sulphuraria* Strain 074G

**DOI:** 10.1371/journal.pone.0148358

**Published:** 2016-02-09

**Authors:** Fean Davisunjaya Sarian, Delicia Yunita Rahman, Otto Schepers, Marc Jos Elise Cornelis van der Maarel

**Affiliations:** 1 Biochemistry Research Division, Faculty of Mathematics and Natural Sciences, Institut Teknologi Bandung, Bandung, Indonesia; 2 Aquatic Biotechnology and Bioproduct Engineering, Engineering and Technology institute (ENTEG), University of Groningen, Groningen, The Netherlands; Universidade Federal de Vicosa, BRAZIL

## Abstract

As a consequence of the inhibition of one of the steps in the biosynthesis of the photopigments chlorophyll and phycobilin, the red microalga *Galdieria partita* excretes coproporphyrinogen III in the medium when growing on glucose. No coproporphyrinogen III was found when the closely related red microalgae *G*. *sulphuraria* strain 074G was grown on glucose and excessive amounts of oxygen. When under the same conditions oxygen was limiting, coproporphyrinogen III was present in the medium. We conclude that not glucose but the amount of oxygen in the medium results in the accumulation of coproporphyrinogen III. This is explained by the inactivition of the oxygen-dependent coproporphyrinogen III oxidase that converts coproporhyrinogen III to protoporphyrinogen IX, one of the intermediate steps in the biosynthesis of chlorophyl and phycobilin.

## Introduction

Chlorophylls (Chl) are the primary absorbing pigment of photosynthetic organisms and are present in the light-harvesting complex as well as in the photosynthetic reaction center. Chl *a* and Chl *b* are the major types of chlorophylls found in photosystems. Chlorophylls are cyclic derivatives of porphyrins (tetrapyrroles) that contain a chelated Mg^2+^ and a characteristic fifth ring [1]. The chlorophyll biosynthetic pathway is well studied in terms of the enzymes involved [2]. The biosynthetic pathway of chlorophyll and heme share a number of common intermediates starting from 5-aminolevulinic acids (ALA) through protoporphyrin IX (PROTO) [1, 3, 4]. The chlorophyll-specific pathway from PROTO starts with the insertion of a Mg^2+^ ion and the last step in the synthesis of Chl *a* and *b* is catalyzed by chlorophyll synthetase [4]. The final step in heme biosynthesis is the insertion of the Fe^2+^ ion in PROTO by ferrochelatase. Heme serves as a cofactor in different cellular processes, for example in oxygen storage and transport, signal transduction, and oxidative metabolism [4]. The chlorophyll and heme biosynthesis pathway share the enzyme coproporphyrinogen III oxidase (CPO) that catalyzes the conversion of coproporphyrinogen III (COPROGEN) to protoporphyrinogen IX (PROTOGEN) [5]. The first report on CPO was by Falk et al. [6] and a few years later Del Battle et al. [7] isolated CPO from rat liver, presenting evidence that this enzyme formed PROTOGEN. CPO has an absolute requirement for molecular oxygen as an electron acceptor for the oxidative decarboxylation of COPROGEN, as described for animals, yeast, and aerobically grown bacteria [8, 9, 10]. A number of genes encoding CPO from several organisms have been cloned and sequenced [11, 12, 13].

Research conducted by Stadnichuk et al. [14] with the extremophilic red microalga *Galdieria partita* grown under mixotrophic and heterotrophic condition showed that on glucose the algal cells changed color from green to yellowish. This color change was attributed to a reduced amount of chlorophyll and phycobilin, a blue-colored photo pigment, in the cells. In addition, it was found that coproporphyrin III (COPRO), the oxidized form of COPROGEN, was excreted into the growth medium coloring it pink. The authors concluded that D-glucose inhibited the biosynthesis of chlorophyll and phycobiliprotein at the transcriptional level, resulting in lower levels of these photo pigments and yellow-colored algal cells and at the same time the excretion of COPRO [14].

The growth of a green mutant of *Galdieria sulphuraria* (strain 074G), phylogenetically very closely related to *G*. *partita* [15], was investigated on glucose and varying oxygen concentrations and the formation of COPRO was monitored. When oxygen was limiting, COPRO production increased with time. While Stadnichuk et al. [14] considered the influence of glucose on the excretion of COPRO, we found a correlation between the production of COPRO and the presence of oxygen. It is therefore proposed that COPRO excretion is directly related to oxygen limitation and only indirectly to the presence of glucose.

## Material and Methods

### Strain and growth medium

The red microalgae *G*. *sulphuraria* 074 [16] was obtained from AlgaeBiotech (Weesp, The Netherlands). Stock cultures were maintained by sub-cultivation in Allen medium without organic carbon substrates under constant light (100 μmol photons m^-2^ s^-1^) on a shaker at 150 rpm and 40°C. Allen’s medium [17] consisted of 1.32 g/L (NH_4_)_2_SO_4_, 0.27 g/L KH_2_PO_4_, 0.25 g/L MgSO_4_·7H_2_O, 0.074 g/L CaCl_2_·2H_2_O, 11 mg/L FeCl_3_, 2.8 mg/L H_3_BO_3_, 1.8 mg/L MnCl_2_, 0.218 mg/L ZnSO_4_·7H_2_O, 0.05 mg/L CuSO_4_, 0.023 mg/L NH_4_VO_3_, and 0.024 mg/L Na_2_MoO_4_·2H_2_O. The pH of medium was adjusted to 2.0 with 4 M H_2_SO_4_ prior to autoclaving at 120°C for 20 min. All chemicals were reagent grade and were obtained from Sigma-Aldrich (Germany). All solutions were prepared with distilled deionized water.

### Growth condition

To prepare the inocula, *G*. *sulphuraria* 074G was cultivated in 250-mL shake flasks with 100 mL of Allen medium pH 2.0 at 40°C, 150 rpm on orbital shakers under continuous illumination provided by day light LED lamps (autotroph condition). Flasks were closed with standard cotton plugs. All incubations were made under continuous illumination at 40°C, unless stated otherwise.

The aerobic and oxygen limited condition cultivation experiments were conducted using 250-mL shake flasks with standard cotton plugs or a sealed lid at the top of the flask, respectively. Fresh media was prepared with 10 g/L glucose. Cultures were inoculated with about 10^5^ autotrophically grown cells per ml. Experiments were carried out in duplicate.

The production of porphyrin-like compound was studied with glucose, galactose, dulcitol, or sucrose as substrate (all 10 g/L). The number of cells at the start was about 10^5^ per mL. The growth of microalgae and production of porphyrin-like compound was monitored by means of measuring the optical density using a spectrophotometer at resp. 800 nm and 400 nm. All experiments were performed in triplicate.

A 1-L bioreactor (Applikon, The Netherlands) was used to monitor porphyrin-like compound production under heterotrophic conditions. Heterotrophic conditions were created by wrapping the bioreactors in aluminium foil to prevent light from entering the reactor and by supplying 10 g/L glucose as a carbon source. Each culture was inoculated with autotrophically-grown microalgae cells an initial OD_800nm_ of about 0.1 and incubated for up to 14 days. The reactors were agitated by a magnetic stirrer at 150 rpm and were sparged with air to create aerobic condition.

A 2-L bioreactor (Applikon, The Netherlands) was used to study the effects of additional air supply on the growth of *G*. *sulphuraria* 074G and porphyrin-like compound production. The pH and temperature in bioreactor were controlled at 2.0 and 40°C, respectively. Each culture was inoculated with about 10^5^ cells per mL. Four different experiments were performed with varing conditions, which are described below. All these cultures were illuminated.

1. *G*. *sulphuraria* 074G was grown mixotrophically with glucose as the carbon source. During the growth, the culture was well aerated by stirring and active air supply.
2. cultures were grown as described above, except the medium was continuously stirred and no additional air was supplied. The dissolved air in the growth medium was the only oxygen supply for the microalga.
3. cultures were initiated as described in condition (ii), except that after the mid of the exponential phase was reached, additional air was supplied by mixing the culture with atmospheric air.
4. cultures were mixed well with air from the start and when the mid of the exponential growth phase was reached, the air supply was shut off, creating oxygen limiting condition.

The cultures were analyzed for growth and porphyrin-like production almost daily for 24 days. The profiles of oxygen concentrations were monitored throughout experiments with an oxygen electrode (Applikon, The Netherlands).

### Sampling and determination of growth

Growth in flasks or the bioreactors was followed by sampling up to 24 days. The absorbance value of the culture was measured with a UV/visible spectrophotometer (Hach Lange DR 3900 RFID, Germany). Absorbance at 400 nm, 618 nm, and 800 nm was used to monitor the porphyrin-like compound production, the amount of phycocyanin, and the cell density, respectively.

Fluorescence intensity measured using excitation wavelength 457 nm (Cobalt Laser 50 mW) and emission scanned from 450 to 1000 nm. Fluorescence was collected and collimated with plano conves mirror (2,5 cm diameter and 15 mm focal length).

## Results

### Porphyrin-like compound

A similar experiment as reported by Stadnichuk, et al. [14] was conducted with the green mutant of a closely related red microalga, *G*. *sulphuraria*. This strain, 074G, is still green when grown in the dark on glucose, as reported by [18]. When strain 074G was grown with light on glucose at 150 rpm in flasks sealed with a standard cotton plug, high cell density was achieved but the medium did not turn pink indicating that no porphyrin-like compound was formed. When this strain was grown under the same conditions, but in sealed flasks thereby creating oxygen-limitation, the medium turned pink after 22 days (data not shown). No pink color was observed when glucose was absent.

**Fig 1:**
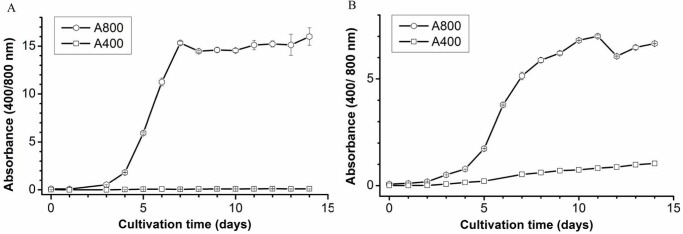
Heterotrophic growth of *G*. *sulphuraria* 074G on glucose and production of the porphyrin-like compound. (A) With and (B) without additional aeration. Absorbance 400 nm: Porphyrin-like compound; Absorbance 800 nm: total biomass. Growth curves were determined in triplicate and symbols represent means.

**Fig 2:**
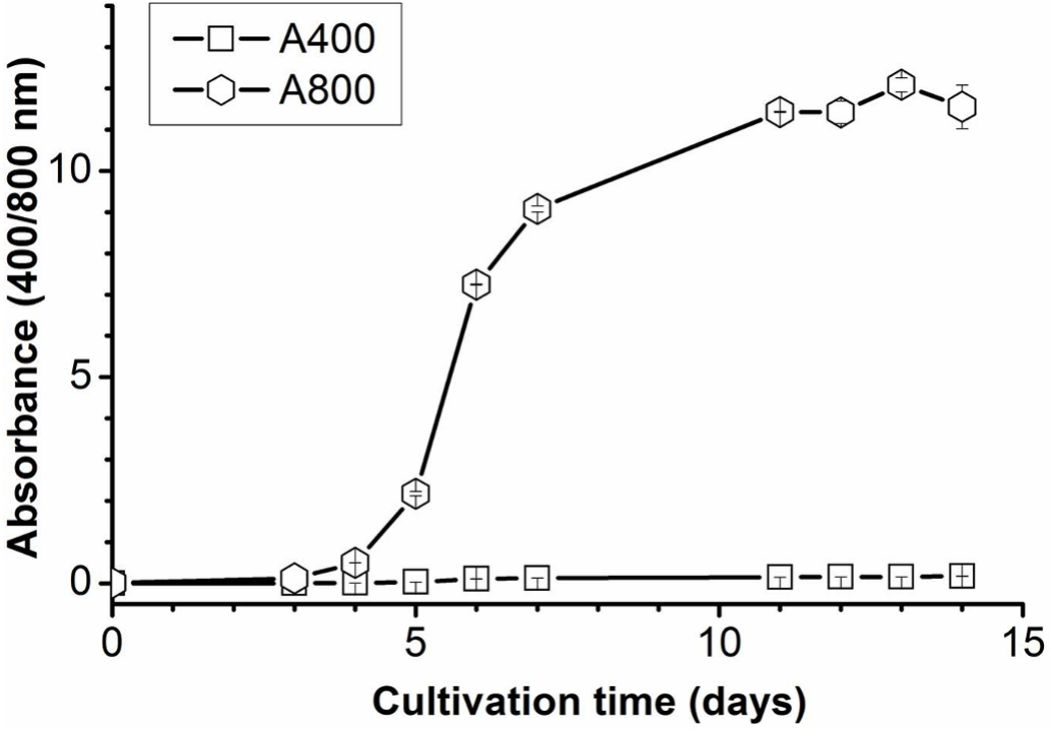
Mixotrophic growth of *G*. *sulphuraria* 074G under active aeration. Absorbance 400 nm: porphyrin-like compound; Absorbance 800 nm: total biomass.

The effect of heterotrophic aerobic conditions (with glucose, air and no light) on the production of the porphyrin-like compound was further investigated using a 1-L bioreactor allowing proper mixing and active oxygen input. The input of additional air during growth of strain 074G resulted in a 2.5-fold increase in biomass compared to the same conditions with no extra oxygen input. Production of the porphyrin-like compound was not detected when strain 074G under these conditions (Fig 1A). However, when the bioreactor was not actively aerated, the growth of strain 074G was much lower. The porphyrin-like compound accumulated in the medium after about 4 days, as a clear absorbance was observed at 400 nm (Fig 1B). When mixotrophic conditions (light, glucose) were applied and the cultures were actively aerated, no porphyrin-like compound accumulated in the growth medium over the course of 14 days, although the cells grew well (Fig 2).

### Confirmation of the nature of the porphyrin-like compound

**Fig 3:**
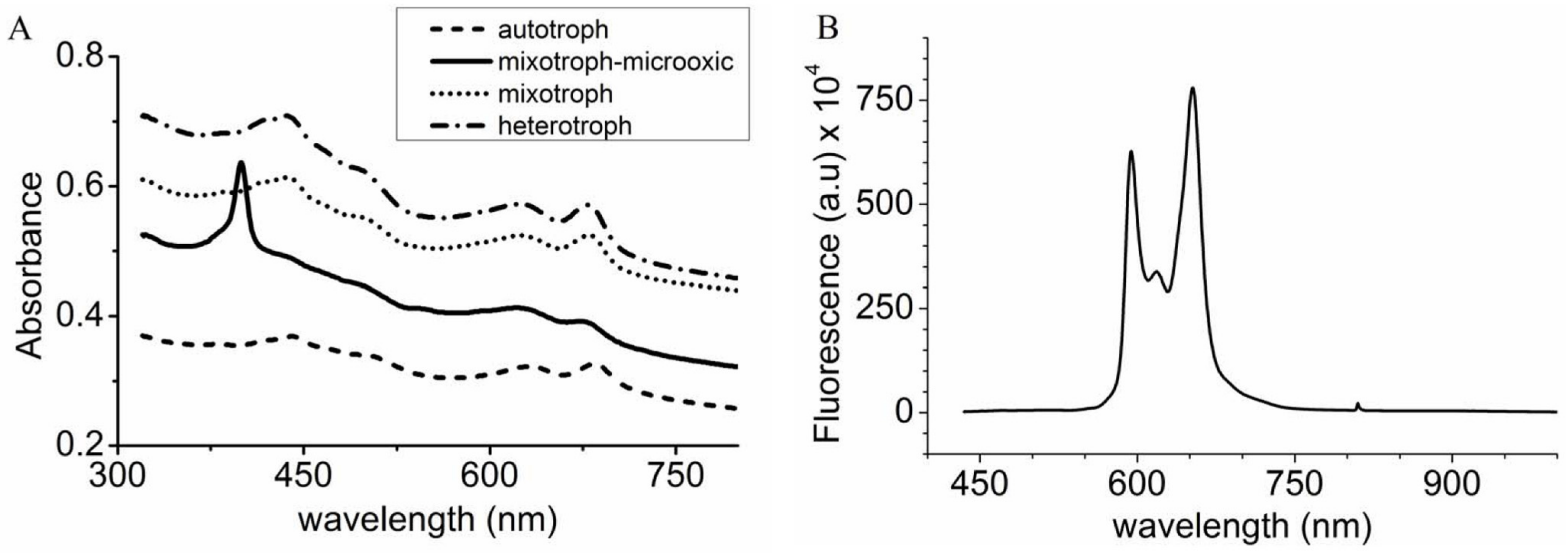
Absorption and fluorescence spectra of *G*. *sulphuraria 074G* whole cells and culture medium. (A) Absorption spectra of *G*. *sulphuraria* 074G cells grown under various conditions. Absorption peak at 680 nm and at 618 nm corresponds to chlorophyll *a* and phycocyanin, respectively, and the absorption peak at 400 nm belongs to a porphyrin-like compound. (B) Fluorescence spectra of the medium at day 24 from *G*. *sulphuraria* 074G grown under mixotrophic condition with limited oxygen amounts. Excitation spectrum at 457 nm, the fluorescence emission spectrum was obtained upon excitation at the maximum of the Soret band.

To determine the nature of the porphyrin-like compound produced by strain 074G, the cultures grown under the following conditions were analyzed spectrophotometrically: autotrophic (light, extra air input); heterotrophic (dark, glucose, extra air input); mixotrophic (light, glucose, extra air input); and mixotrophic-microoxic (light, glucose, no air input). A single peak centered near 400 nm was found for mixotrophic-microoxic conditions but not for the other growth conditions (Fig 3A). The fluorescence spectrum of the culture medium of the mixotrophic-microoxic conditions showed two major emission peaks, a primary maximum at 594 nm and a secondary maximum at 652 nm (Fig 3B).

### The production of porphyrin-like compound is influenced by oxygen availability

**Fig 4:**
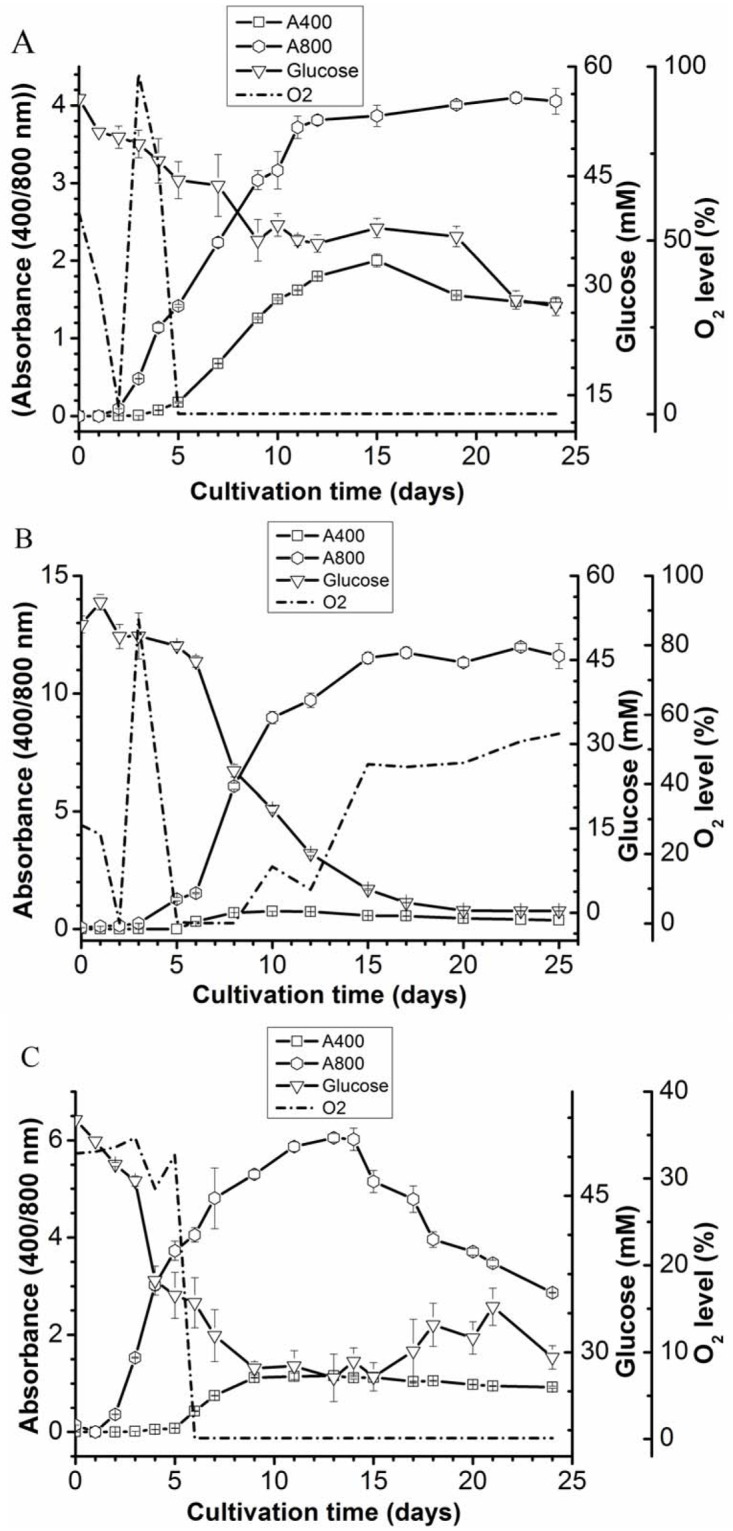
Effect of oxygen limitation on the porphyrin-like compound production and growth of *G*. *sulphuraria* 074G. Absorbance 400 nm: Porphyrin-like compound; Absorbance 800 nm: total biomass. (A) Following inoculation, the cultures were incubated mixotrophically without additional air supply. (B) Growth of *G*. *sulphuraria* 074G under an initial air supply rate of 5 L/min and termination of the air supply at the mid-exponential phase (day 5); (C) Growth when the active aeration of the culture was switched on at the mid-exponential phase (day 5).

To better understand the effect of air/oxygen on the accumulation of the porphyrin-like compound, the amount of oxygen present in the medium under different growth conditions was measured. Cells of strain 074G did not grow well under mixotrophic conditions (glucose, light) without active air supply. However, the pink color was detectable when the dissolved oxygen concentration dropped below detection limits (day 5, Fig 4A). To understand the relationship between oxygen concentrations and the production of the porphyrin-like compound in more detail, the active aeration of the culture was either switched on or off when the culture was at the mid-exponential growth phase. Starting with no aeration, thus creating oxygen-limiting conditions from the start, the medium turned slightly pink after 5 days when also the oxygen levels dropped to zero. The amount of pink color leveled of when the aeration was turned on at day 7, the time at which the culture was in the mid-exponential phase (Fig 4B). Switching off the aeration in the middle of the exponential growth phase gave the opposite effect. Now an intense pink color developed as the oxygen levels approached zero (Fig 4C)

To explore whether the porphyrin-like compound is also formed on substrates other than glucose, strain 074G was grown on dulcitol, galactose and sucrose using tightly sealed flasks creating oxygen-limiting conditions. Dulcitol cultures produced a similar amount of porphyrin-like compound as glucose cultures, while galactose and sucrose cultures had only about 35% and 16% of the amount of the porphyrin-like compound, respectively, when compared to glucose (Table 1).

**Table 1:** Effect of various carbon sources on the production of the porphyrin-like compound.

Substrate (1% (w/v))	A400	A800	Ratio A400/A800
Glucose	1.02 ± 0.004	7.00 ± 0.28	0.15
Galactose	0.35 ± 0.02	7.68 ± 0.33	0.05
Dulcitol	0.90 ± 0.05	8.67 ± 0.31	0.10
Sucrose	0.16 ± 0.00	8.05 ± 0.67	0.02

## Discussion

In an earlier report of Stadnichuk et al. [14], it was stated that the synthesis of chlorophyll and phycobiliprotein in the red microalgae *Galdieria partita* is inhibited by glucose, leading to the accumulation of COPROGEN in the cells and finally in the growth medium. The cellular contents of Chl *a* and phycocyanobilin clearly decreased under heterotrophic or mixotrophic conditions. The inhibition of the synthesis of chlorophyll by glucose is also reported for the protist *Euglena gracilis* [19] and the red microalga *Cyanidium caldarium* [20]. In the present study, *G*. *sulphuraria* strain 074G was grown on glucose under similar condition as Stadnichuk and coworkers [14] used for *G*. *partita* but no COPROGEN was found in the medium. However, when strain 074G was grown on glucose under oxygen limiting conditions the medium clearly turned pink. The fluorescence spectrum of the pink compound showed a peak at 594 nm and 652 nm (Fig 3B), indicating that coproporphyrin III (COPRO), the oxidized form of COPROGEN, was formed [14]. The UV-Vis spectrum of whole cells grown on glucose under oxygen limiting conditions gave a maximum at 400 nm, the Soret peak [21], and minor peaks in the 600–650 nm range (Fig 3A), pointing at production of phycocyanin [22] and thus a functioning chlorophyll pathway.

The correlation between oxygen limitation and the production of COPRO was much more prominent when strain 074G was grown in a well-mixed bioreactor with or without active aeration (Fig 1A and 1B). The ultimate proof that not glucose but the oxygen concentration leads to the accumulation of COPRO came when the amount of oxygen in the medium of a well-mixed, (not) aerated culture growing on glucose was measured. The cultures with oxygen and glucose did not produce COPRO (Fig 4B), while COPRO accumulated in the medium of in cultures with glucose without oxygen (Fig 4A and 4C). Further proof that not glucose but oxygen results in accumulation of COPRO was obtained when growing strain 074G on other sugars without active air supply (Table 1).

**Fig 5:**
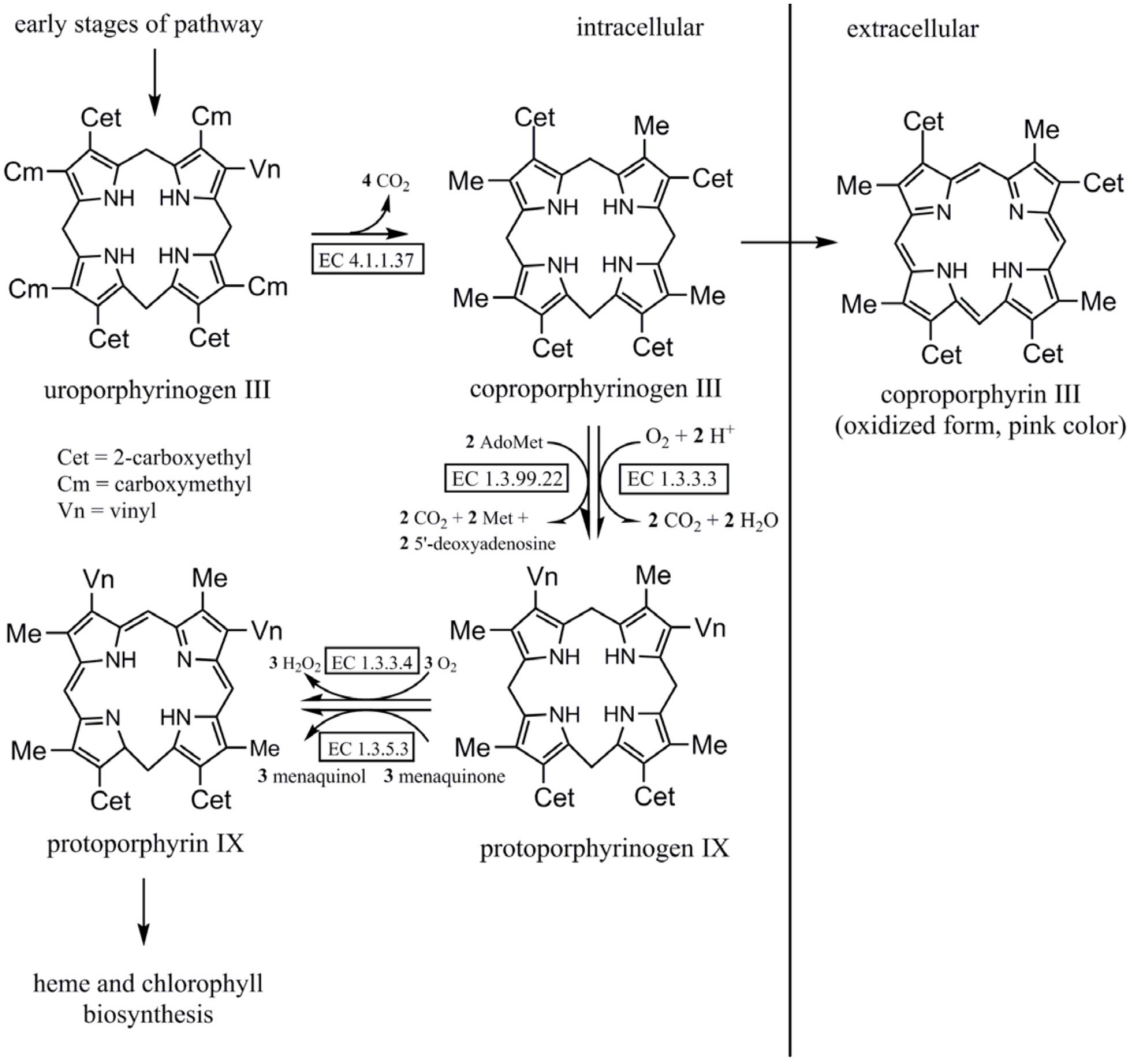
Later stages of the biosynthetic pathway of porphyrin. The enzymes catalyzing each step are indicated (adapted from IUBMB) [23].

The biosynthesis of chlorophyll starts with 5-aminolevulinic acid (ALA) and proceeds through a number of pophyrin/porphyrinogen derivatives (Fig 5) [1]. COPROGEN is converted to protoporphyrinogen IX by two structurally unrelated oxygen oxidoreductases (coproporphyrinogen III oxidase, CPO), the oxygen-dependent CPO, HemF (E.C. 1.3.3.3) and oxygen-independent CPO, HemN (1.3.99.22) (Fig 5).

HemF is only active when oxygen is present, while HemN requires *S*-adenosylmethionine (SAM) for catalysis [24]. The distribution of those two enzymes in various organisms is very heterogeneous. HemF is commonly found in eukaryotic organisms and less frequently in prokaryotes, while HemN is conserved widely in prokaryotic organisms [25]. However, some photosynthetic bacteria such as *Rhodobacter sphaeroides* and *Synechocystis* sp. carry the genes encoding both enzymes, which are differently utilized to thrive in habitats with fluctuating oxygen tensions [24, 26]. Interestingly, the genes encoding HemF and HemN are both found in *G*. *sulphuraria*, which have been submitted as Gasu_19740 [27] and Gasu_49660 [28] for oxygen-dependent CPO and oxygen-independent CPO, respectively.

Assuming that both genes are operational and expressed, the results reported in this communication can be explained as follows. As Falk and colleagues [29] have shown, HemF is completely inhibited by anaerobic condition. The same was observed for the HemF of *Escherichia coli*; under anaerobic condition this CPO failed to convert COPROGEN [10]. When strain 074G experiences oxygen limiting conditions, the HemF enzyme does not function properly and COPROGEN accumulates in the cells and at certain moment ends up in the growth medium (Figs 1 and 4). As the pH of the medium is low, the COPRO stays in solution and colors the medium pink [14]. The fact that still some chlorophyll is produced by strain 074G is most likely due to the activity of HemN, the oxygen-independent enzyme.

The observation by Stadnichuk, et al. [14] that the presence of glucose results in the accumulation of COPRO can be explained by a lack of oxygen in the culture. They grew *G*. *partita* in flasks but it is nowhere stated if these cultures were mixed or not. We therefore assume that these cultures experienced oxygen limitation, leading to the accumulation of the COPRO as we found when we used flasks with tight sealing. All of the oxygen that is present is used by the microalgae to support the conversion of glucose into energy by glycolysis, the Krebs cycle, and the oxidative phosphorylation, leaving no oxygen for the oxygen-dependent oxidoreductase and thereby rendering this enzyme inactive.

## Conclusion

Oxygen limitation and not glucose leads to an impaired conversion of coproporphyrinogen III and thus accumulation of it in the culture medium. The impaired conversion can be explained by a lack of oxygen, which is required for the oxygen-dependent coproporphyrinogen III oxidase enzyme.
